# Online Health Resource Use by Individuals With Inflammatory Bowel Disease: Analysis Using the National Health Interview Survey

**DOI:** 10.2196/15352

**Published:** 2020-09-24

**Authors:** Rong Yin, David M Neyens

**Affiliations:** 1 Department of Industrial Engineering Clemson University Clemson, SC United States

**Keywords:** internet, searching behavior, access to information, inflammatory bowel disease, logistic regression model, National Health Interview Survey

## Abstract

**Background:**

The internet has enabled convenient and efficient health information searching which is valuable for individuals with chronic conditions requiring some level of self-management. However, there is little research evaluating what factors may impact the use of the internet for health-related tasks for specific clinical populations, such as individuals with inflammatory bowel diseases.

**Objective:**

Our goal was to investigate the factors that influence internet use in acquiring health information by individuals with inflammatory bowel diseases. Specifically, we identified factors associated with internet searching behavior and using the internet for completing health-related tasks.

**Methods:**

We used 2016 National Health Interview Survey weighted data to develop logistic regression models to predict the likelihood that individuals with inflammatory bowel diseases would use the internet for 2 types of tasks: seeking health information through online searches and using the internet to perform health-related tasks including scheduling appointments and emailing care providers.

**Results:**

2016 National Health Interview Survey weighted data include more than 3 million weighted adult respondents with inflammatory bowel diseases (approximately 1.29% of adults in the weighted data set). Our results suggest that approximately 66.3% of those with inflammatory bowel diseases reported using the internet at least once a day, and approximately 14.7% reported being dissatisfied with their current health care. About 62.3% of those with inflammatory bowel diseases reported that they had looked up health information online, 16.3% of those with inflammatory bowel diseases reported that they had scheduled an appointment with a health care provider online, and 21.6% reported having used a computer to communicate with a health provider by email. We found that women who were self-regulating their care were more likely to look up health information online than others. Both middle-aged and older adults with inflammatory bowel diseases who were unsatisfied with their current health care were less likely to look up health information online. Frequent internet users who were worried about medical costs were more likely to look up health information online. Similarly, the results from our statistical models suggest that individuals with inflammatory bowel diseases who were frequent internet users were more likely to use the internet for specific health-related tasks. Additionally, women with inflammatory bowel diseases who reported being married were less likely to use the internet for specific health-related tasks.

**Conclusions:**

For those with inflammatory bowel diseases, there are additional socioeconomic and behavioral factors that impact the use of the internet for health information and health-related tasks. Future research should evaluate how these factors moderate the use of the internet and identify how online resources can support clinical populations in ways that improve access to information, support health self-management, and subsequently improve health outcomes.

## Introduction

### Background

The internet is seen as a reliable alternative source of health information [1,2], and people seek health information online to gain additional information about health conditions or procedures [3], as well as to discuss their specific condition and health status through online discussion groups [4]. The internet may provide a convenient method for patients to obtain health information regardless of geographical restrictions [5-7] or access to care providers. Past research [8-12] has found that using the internet to search health information leads to better health outcomes, and the internet is believed to be a good source of health information to support developing health knowledge, ongoing long-term self-management of care, and monitoring the condition of patients. Research [13] has found that most people use the internet to acquire specific information regarding their own health status or that of their family or friends.

Individuals with chronic diseases are a unique user population in terms of their potential use of online health information in self-management of their health. The prevalence of chronic diseases is high in the United States; Ward et al [14] reported that nearly 50% of adults have one chronic disease, and 25% have multiple conditions. Past research suggests that searching health information online may be a common behavior for people with chronic health conditions [15] and that online information seekers’ health literacy and engagement may correlate with their ability to manage their chronic health conditions [16]. It has been shown that individuals with chronic diseases are more willing to search health information on the internet than those without such conditions [17]. In addition, patients who have chronic diseases but who do not have health insurance are more willing to search for health information on the internet than individuals with insurance [17], supporting results from other studies [3,18] that suggest that the involvement and motivation of users impact their engagement in online health information searching, with highly motivated users, such as those with chronic diseases, applying more effort in the information searching task. Additionally, there are multiple factors, including a person’s gender, age, and socioeconomic status that influence an individuals’ online information searching behavior and internet usage [1,15,19-23].

To ensure the effectiveness of the internet related to health information, the US Department of Health and Human Services [24] has provided design guidelines to improve the user experience of individuals with various levels of health literacy, paying special attention to people with limited abilities. Not only are those with low health literacy less likely to use the internet for information searching and emailing [25], they are also more likely to forget information and experience working memory overload when interacting with websites [24] compared to internet users with higher health literacy. These users have been found to spend 9 times longer conducting information searching tasks than higher literacy users, and they tend to read word by word rather than glancing at the entire page for the more relevant information [26]. In addition, there are other barriers for all online health information seekers including limited accessibility to the content published in research journals, the complexity of the clinical language used, and the inability to evaluate the reliability of health information websites [16]. Lee et al [16] argue that these barriers could be reduced by increasing the involvement of health professions in guiding the health information seeking process and improving general health literacy.

### Crohn Disease and Ulcerative Colitis

Crohn disease and ulcerative colitis are collectively referred to as inflammatory bowel disease (IBD) [27,28], a chronic condition that affects the intestines, colon, and bowel [29]. It is a complex, incurable disease [30] that can result in long-term disability or mortality [31], and its highest incidence occurs in younger adults [31,32]. A recent study [33] suggested that the incidence of IBD has seen a dramatic increase to over 0.3% in North America and many European countries, and the incidence of IBD is expected to continuously increase [31].

Generally, the majority of studies related to IBD focus on its pathology and medical treatment. Although some studies have focused on the diagnosis of IBD [29], predictors of its disabling consequences [34], its pathogenesis [27,28,35,36], and the dietary habits of those with IBD [37,38], few have examined which factors may influence individuals with IBD to search the internet for health care–related information. Yet, the management of IBD depends on self-management of the disease and a level of health literacy. It has been found that many health websites did not provide appropriate coverage of prognoses, side effects, and additional health risks associated with IBD but did cover symptoms, complications, and treatment options [39]. Additionally, it was reported that information related to self-management of IBD was not widely included in health websites [39], and thus the use of online search behavior associated with IBD is an important area of research.

### Research Objectives

The overall objective of this study was to investigate the factors that influence the use of the internet to acquire health information for individuals with IBD. We examined 2 types of internet-related activities: searching the internet for health information and using the internet for health-related tasks such as scheduling appointments with health care providers and communicating with a health care provider by email. We evaluated a number of potential factors that might impact how an individual with IBD uses the internet for health information. Previous research has shown that a number of factors impact internet usage for health information in general populations including: gender [5,6,40,41], age [40,41], level of education [42], health literacy [25], health insurance coverage [17], and level of income [19,23].

## Methods

### Data Source: National Health Interview Survey

The National Health Interview Survey (NHIS), which is conducted by the National Center for Health Statistics, covers broad health topics [43]. The data that are collected are weighted to represent the general population of the United States. The topics and the questions in the survey have evolved over time, and thus, the type of data collected each year varies. The 2016 NHIS [44] included questions asking respondents to self-identify as having IBD (Crohn disease and ulcerative colitis). For our study, several variables in the original data were recoded and combined to form categories to support the analysis and interpretation of the results of our statistical models. The original variable names in the NHIS data files are included in parentheses to facilitate an understanding of how we coded and used the data.

### Dependent Variables

This study focused on the behaviors and experiences, during the year preceding the interview, of adult individuals who reported having IBD (ULCCOLEV). The dependent variables in this study were related to internet usage: (1) individuals searching for health information on the internet (HIT1A), (2) individuals using the internet to schedule appointments with health care providers (HIT3A), and (3) individuals using the internet to communicate with health care providers via email (HIT4A). All dependent variables were recoded as binary variables (1, they reported that they had done the activity in the previous 12 months; 0; they had not).

### Independent Variables

Demographic variables such as sex (SEX) and age (AGE_P) were used in the analysis. The age variable was recoded into 3 groups: younger adults (18-35 years old), middle-age adults (36-55 years old), and older adults (older than 55). We recoded marriage status (R_MARITL) as a binary variable (1, married; 0, not married) where not married included never married, divorced, widowed, separated, as well as preferred not to answer and nonresponses. Parental status (PAR_STAT) of participants was recoded as being a parent of a child or not a parent of a child. Work status (DOINGLWA) of participants was recoded as employed or not employed.

It is possible that individuals with multiple chronic conditions may use the internet differently than those with a single chronic condition because of the complexity of managing multiple conditions. It is possible that they may receive conflicting medical advice for diverse chronic conditions [45,46]. Therefore, 7 other chronic conditions were also included in our analysis as binary variables: hypertension (HYPEV), high cholesterol (CHLEV), coronary heart disease (CHDEV), asthma (AASMEV), cancer (CANEV), diabetes (DIVEV1) and chronic/long-term liver conditions (LIVEV).

Other variables that may impact an individual’s online information searching behaviors were also included in the analysis such as socioeconomic considerations, the level of satisfaction with health care services, and internet usage frequency. Whether the respondent reported having trouble finding a care provider in the previous 12 months (APRVTRYR) was recoded as reported trouble in finding a care provider and reported no trouble in finding a care provider. The respondents who reported being worried about paying medical bills (AWORPAY) were recoded as worried and not worried, with the former category including those who were very worried and those who were somewhat worried. A new variable was created to indicate whether participants were self-regulating care in a number of possible ways. This self-regulating care included whether the respondents reported doing at least one of the following actions: skipping medication doses (ARX12_1), taking less medicine (ARX12_2), delaying filling a prescription (ARX12_3), asking a doctor for less expensive medication (ARX12_4), and using alternative therapies (ARX12_6). A binary variable was created to identify whether the participants reported having seen or talked to a general practitioner in the prior year (AHCSYR9). A variable was also created to determine whether the participants tried to purchase health insurance directly in the prior 3 years by combining the 2 relevant variables of “Tried to purchase health insurance directly” (AINDINS2) and “Purchased health insurance directly” (AINDPRCH). The satisfaction of participants in their health care (ASISATHC) was recoded as satisfied and not satisfied, with the satisfied category including those who reported being very or somewhat satisfied with their health care services. A variable was created identifying frequent internet users based on the respondent’s frequency of internet usage (AWEBOFNO and AWEBOFTP). Frequent internet users were identified as such if the internet was used at least once a day (ie, at least 7 times per week) and were classified as not frequent internet users otherwise.

### Statistical Analysis

The data were analyzed using R (version 3.5.0). Specifically, the svyglm function (Survey package; version 3.34) [47] was used for logistic regression, and stepwise deletion was used to remove insignificant parameters from the model in order to identify the best model for each dependent variable. As the weighted sample size was large, α=.01 was used to assess significance.

## Results

### Descriptive Statistics

After applying the data weights, the sample size of individuals who reported having IBD was 3,155,477 (approximately 1.29% of all the adults in the weighted data set); approximately 64.4% (2,032,022) of the respondents were female, the average age of the respondents was 52.8 (SE 0.87) years, and approximately 49.9% of the respondents (1,575,168) reported being married. Approximately 80.7% (2,544,995/3,155,477) of the respondents reported having seen or talked to a general practitioner in the previous year, with very few (273,977/3,155,477, 8.7%) reporting having trouble finding a provider in the previous 12 months, although 14.7% (464,376/3,155,477) reported being dissatisfied with their health care. Approximately 42.6% (1,344,253/3,155,477) and 41.2% (1,288,836/3,155,477) of the respondents also reported having hypertension or high cholesterol, respectively, which were the 2 highest prevalences of comorbidities examined for individuals who had IBD. More than half of the respondents (1,965,639/3,155,477, 62.3%) reported looking up health information online, and approximately 66.3% (2,090,505/3,155,477) reported being frequent internet users, using it at least daily. In terms of the health-related tasks, 16.3% (515,253/3,155,477) of those with IBD reported scheduling an appointment with a health care provide online, and 21.6% (680,872/3,155,477) reported having used computer to communicate with a health provider by email. The complete demographic information of the respondents is in Table 1.

**Table 1:** The characteristics of the sample of survey respondents who reported having IBD.

Variable			Weighted, n (%)
**Age**			
	Younger adults (18-35 years old)	454,950 (14.4)	
	Middle age adults (36-55 years old)	1,159,430 (36.7)	
	Older adults (>55 years old)	1,541,097 (48.8)	
**Sex**			
	Male	1,123,455 (35.6)	
	Female	2,032,022 (64.4)	
Married			1,575,168 (49.9)
Employed			1,548,101 (49.1)
Has at least one child			670,310 (21.2)
Looked up health information online			1,965,639 (62.3)
Used computers to schedule an appointment with a health care provider			515,253 (16.3)
Used computer to communicate with a health care provider by email			680,872 (21.6)
Reported having hypertension			1,344,253 (42.6)
Reported having high cholesterol			1,298,836 (41.2)
Reported having coronary heart disease			320,715 (10.2)
Reported having asthma			636,538 (20.2)
Reported having cancer			491,356 (15.6)
Reported having diabetes			564,795 (17.9)
Reported having chronic/long-term liver conditions			127,679 (4.0)
Reported having trouble in finding a provider in the previous 12 months			273,977 (8.7)
Reported being worried about paying medical bills			1,732,203 (54.9)
Reported multiple types of self-regulating care			1,192,446 (37.9)
Reported having seen or talked to a general doctor in the previous year			2,544,995 (80.7)
Reported trying to purchase health insurance directly in the previous 3 years			426,541 (13.5)
Reported being unsatisfied with their health care			464,376 (14.7)
Used the internet frequently (at least daily usage)			2,090,505 (66.3)
Reported being worried about medical costs			1,618,723 (51.3)

### Looking Up Health Information on the Internet

A binary logit model was created to evaluate how individuals with IBD use the internet for information seeking (Table 2). Among the individuals with IBD, those who also had asthma were more likely to look up health information online compared to others (adjusted odds ratio [OR] 2.97, 99% CI 1.17 to 7.54). Although several different types of chronic conditions were initially included in the model, only the variable indicating asthma was a significant predictor impacting the likelihood of those with IBD looking up health information online.

Both middle-aged and older women were less likely to look up health information online compared to others (adjusted OR 0.07, 99% CI 0.004 to 0.96 and adjusted OR 0.02, 99% CI 0.001 to 0.29, respectively). Women with IBD who reported self-regulating care were more likely to look up health information online than others (adjusted OR 9.87, 99% CI 1.49 to 65.37). Both middle-aged (36-55 years old) and older (over 55 years old) adults who were married were more likely to look up health information online (adjusted OR 22.20, 99% CI 1.46 to 336.97 and adjusted OR 23.81, 99% CI 1.75 to 327.01, respectively). Both middle-aged and older adults who were unsatisfied with their current health care were less likely to look up health information online (adjusted OR 0.03, 99% CI 0.002 to 0.58 and 0.03, 99% CI 0.001 to 0.71, respectively). Individuals who were employed and were unsatisfied with their current health care were less likely to look up health information online (adjusted OR 0.07, 99% CI 0.007 to 0.62). Additionally, frequent internet users who were worried about the medical costs of an illness/accident were more likely to look up health information online (adjusted OR 12.18, 99% CI 2.08 to 72.24).

**Table 2:** Binary logit model for the likelihood of looking up health information on internet.

Parameter	Estimate	99% CI	SE	*t* value	*P* value	Adjusted OR^a^	99% CI
Intercept	–2.95	(–4.91, –0.99)	0.76	–3.87	<.001	0.05	(0.007, 0.37)
Female	3.08	(0.75, 5.42)	0.91	3.40	.001	21.76	(2.12, 225.88)
Middle-aged adults	0.98	(–1.11, 3.08)	0.81	1.21	.228	—^b^	
Older adults	1.43	(–0.59, 3.44)	0.78	1.83	.068	—	
Married	–2.72	(–5.03, –0.42)	0.90	–3.04	.002	0.07	(0.007, 0.66)
Employed	0.95	(–0.06, 1.95)	0.39	2.42	.016	—	
Had asthma	1.09	(0.16, 2.02)	0.36	3.02	.003	2.97	(1.17, 7.54)
Self-regulating care	–1.30	(–2.72, 0.13)	0.55	–2.34	.019	—	
Unsatisfied with health care	4.15	(1.08, 7.22)	1.19	3.49	.001	63.52	(2.94, 1366.49)
Worried about medical costs of illness/accident	–1.30	(-2.57, -0.02)	0.50	–2.62	.009	0.27	(0.08, 0.98)
Frequent internet users	2.60	(1.47, 3.73)	0.44	5.92	<.001	13.42	(4.35, 41.68)
Female × middle-aged adults	–2.72	(–5.40, –0.04)	1.04	–2.62	.009	0.07	(0.004, 0.96)
Female × older adults	–3.91	(–6.59, –1.23)	1.04	–3.76	<.001	0.02	(0.001, 0.29)
Female × self-regulating care	2.29	(0.40, 4.18)	0.73	3.12	.002	9.87	(1.49, 65.37)
Middle-aged adults × married	3.10	(0.38, 5.82)	1.06	2.93	.004	22.20	(1.46, 336.97)
Older adults × married	3.17	(0.56, 5.79)	1.01	3.13	.002	23.81	(1.75, 327.01
Middle-aged adults × unsatisfied with health care	–3.51	(–6.47, –0.55)	1.15	–3.06	.002	0.03	(0.002, 0.58)
Older adults × unsatisfied with health care	–3.48	(–6.61, –0.34)	1.22	–2.86	.004	0.03	(0.001, 0.71)
Employed × unsatisfied with health care	–2.72	(–4.97, –0.48)	0.87	–3.12	.002	0.07	(0.007, 0.62)
Worried about medical costs of illness/accident × frequent internet users	2.50	(0.73, 4.28)	0.69	3.64	<.001	12.18	(2.08, 72.24)

^a^OR: odds ratio.

^b^No statistically significant differences were found at α=.01.

### Using Computers to Schedule an Appointment With a Health Care Provider

A binary logistic regression model was created to predict the likelihood that an individual with IBD used a computer to schedule an appointment with their care provider (see Table 3). Those who reported self-regulating their care were more likely to use the internet to schedule an appointment with a provider than those who did not self-regulate (adjusted OR 2.61, 99% CI 1.05 to 6.49). Those who were frequent internet users were more likely to use the internet to schedule an appointment with a provider than nonusers or infrequent users (adjusted OR 15.18, 99% CI 3.56 to 64.72). Women who reported being married were less likely to use the internet to schedule an appointment with a provider (adjusted OR 0.07, 99% CI 0.007 to 0.75).

**Table 3:** Binary logit model for the likelihood of using the internet to schedule an appointment with a health care provider.

Parameter	Estimate	99% CI	SE	t value	*P* value	Adjusted OR^a^	99% CI
Intercept	–5.82	(–8.23, –3.42)	0.93	–6.24	<.001	0.003	(<0.001,0.03)
Female	1.84	(–0.12, 3.79)	0.76	2.42	.016	—^b^	—
Married	2.10	(0.09, 4.11)	0.78	2.69	.007	8.17	(1.09,60.95)
Self-regulating care	0.96	(0.05, 1.87)	0.35	2.72	.007	2.61	(1.05,6.49)
Frequent internet users	2.72	(1.27, 4.17)	0.56	4.82	<.001	15.18	(3.56,64.72)
Female × married	–2.60	(–4.92, –0.29)	0.90	–2.90	.004	0.07	(0.007,0.75)

^a^OR: odds ratio.

^b^No statistically significant differences was found at α=.01.

### Using Email to Communicate With a Health Care Provider

A binary logistic regression model was created to predict the likelihood that an individual with IBD used email to communicate with their care provider (see Table 4). Those who were frequent internet users were more likely to report using email to communicate with a provider (adjusted OR 8.41, 99% CI 3.22 to 21.76). Women who reported being married were less likely to report using email to communicate with a care provider than others (adjusted OR 0.15, 99% CI 0.02 to 0.93).

**Table 4:** Binary logit model for the likelihood of emailing a health care provider.

Parameter	Estimate	99% CI	SE	t value	*P* value	Adjusted OR^a^	99% CI
Intercept	–4.02	(–5.60, –2.43)	0.61	-6.54	<.001	0.02	(0.003,0.09)
Female	1.36	(–0.10, 2.83)	0.57	2.41	.017	—^b^	—
Married	1.42	(–0.07, 2.91)	0.58	2.45	.014	—	—
Frequent internet users	2.13	(1.17, 3.08)	0.37	5.75	<.001	8.41	(3.22,21.76)
Female × married	–1.88	(–3.69, –0.07)	0.70	–2.67	.008	0.15	(0.02,0.93)

^a^OR: odds ratio.

^b^No statistically significant difference was found at α=.01.

## Discussion

### Principal Findings

Our study examined the use of the internet by individuals with IBD to seek health information and to perform health-related activities. The population of interest was examined because these chronic conditions are often self-managed [48], and for those with IBD, understanding their own chronic conditions, experiences, and psychosocial factors can be a critical aspect of their treatment process [49]. Therefore, information acquisition and use are vital for those with chronic conditions to be able to self-regulate their health conditions [50].

In general, previous studies [5,40-42,51] suggest that the gender and age of individuals impact their internet usage for health information. In our model, women who self-regulated their care were more likely to look up health information online. Whereas, women in the middle-age and older age groups were both less likely to look up health information online. It has been suggested that younger individuals are more likely to use the internet than older individuals [49], and the same may be true for using the internet for health information seeking. Future research should continue to examine how the gender and age interaction influence searching for health information on the internet. The main effect of age was not significant in our study which is inconsistent with the findings of previous studies [42,50]. This may due to the fact that we defined age as a 3-level categorical variable (younger adults, middle-age adults, and older adults) and not as a continuous variable. Future studies could examine the impact of age as a continuous variable on the internet usage by individuals with specific chronic conditions including those with IBD.

As the literature suggests, individuals in poor health tend to use the internet more frequently than healthy individuals to look up health information [5,52,53]. Previous research [15,17] has suggested that individuals with multiple chronic health conditions are more likely to use the internet to acquire information with the expectation that it will help improve their condition. Our results suggest that individuals who reported having asthma in addition to IBD were more likely to use the internet for health care information searching. No other comorbidities were significant predictors in our models. Future research should more comprehensively examine comorbidity categories and types to identify if the results for IBD mirror those from previous studies [15,17].

Those who reported self-regulating their care were more likely to use the internet to schedule appointments with health care providers. Additionally, women who self-regulated their care were more likely to look up health information on the internet. This may relate to the fact that those who self-regulated care may utilize these online resources as part of their self-regulating behaviors, for example, searching for suggestions to support self-regulating their care through self-medicating [7]. There are a number of potential reasons that an individual self-regulates care, such as trying to avoid medication side effects or trying to switch to alternative medication or treatment plans [54]. This type of behavior is critically important for individuals with IBD as self-management is a major aspect of the treatment plans [55]. Future work should further evaluate the underlying mechanisms that lead to individuals choosing to self-regulate their care and how the design of health information and internet-supported health tasks support those types of behaviors. Additionally, being dissatisfied with health care has been shown to influence the likelihood of using the internet for health information seeking [56,57]. Our study suggests those who were unsatisfied with their current health care and who were employed were less likely to look up health information online, the same was true for middle aged and older adults who were unsatisfied with their current health care. This may also relate to different information needs when trying to find a reasonable alternative treatment plan or trying to switch health providers [54].

Identifying factors that might impact the use of the internet for health-related tasks and health information searching can identify demographic and specific issues that might lead to targeted interventions and an examination of how online information is designed for and presented to these populations. According to Kittler et al [58], in 2004, 38% of physicians exchanged emails with their patients regularly, and Hobbs et al [59] found that approximately 37% of patients would have agreed to pay out of pocket to be able to communicate with their physicians by email. The estimates of email communication rates with health care providers are likely much higher today than in 2004. In fact, in 2015, a study of patient email communication with health providers suggested that the email use rate ranged from 18.7% to 50.7% among in 14 European countries and that men were found to be more likely to email health providers than women [41]. In our study, we found that 21.6% had emailed a health provider and that those who were frequent internet users were more likely to use email to communicate with their doctors, whereas married women with IBD were less likely to use email in this way. Future research should evaluate if there are other factors that impact the use of these services.

As expected, frequent internet users were shown to be more likely to use the internet to seek health information, schedule an appointment, and email health providers. In this study, we categorized frequent internet users as individuals who used the internet at least daily, yet many people currently use the internet on a more constant basis, and this variable may not capture differences between daily users and more constant users of the internet. Future research should more specifically examine the impact of internet usage frequency on how individuals with IBD use the internet for health care related activities. It would also be interesting to examine the frequency of internet use as a continuous variable and how that would impact the estimates of using the internet for health care tasks for those with IBD.

### Limitations

There are several limitations of this study that should be addressed in future research. The focus of the NHIS survey was not specifically related to the use of the internet for health care–related tasks, nor was it specifically focused on individuals with IBD. Future work could specifically focus on this clinical population and on specific internet-related tasks. Additionally, with the frequent changes to health IT and in the adoption of health technology, it is possible that this survey did not capture some of the specific uses of technology for health-related purposes or possible technologies (eg, smartphones and health-related apps). There may also be other factors that influence the use of the internet for health-related activities that were not captured by the survey, and thus, were not included in this analysis. For example, some insurance companies require that their customers refill their medications online, a situation not captured by the survey. Nor were socioeconomic variables related to internet access included. Additionally, there are other factors that may impact the use of the internet in conducting health-related tasks (eg, mental health comorbidities, cognitive abilities, health literacy skills [60], complexity of the information search tasks, and credibility of target website [61]) that should be evaluated in future studies. The specific underlying mechanisms for self-regulating care, the way self-regulating care can be defined and implemented, and other related behaviors should be evaluated in future research.

In addition, to facilitate this analysis, most of the survey responses were categorized into binary variables that combined some answers with nonanswers and “I don’t know” responses. For example, internet use was transformed into a binary variable of frequent internet use versus infrequent internet use. These dichotomized variables may impact the findings associated with specific variables. Thus, future research could also examine the variables on a broader continuum in order to identify any additional nuances in the data. Additionally, future research should use different methods to identify why some relationships between variables were significant and also to identify the underlying causes so that future information strategies account for these differences and leverage what we know about the individuals with IBD and their internet health-related behaviors.

### Conclusions

As the use of health information technology increases and evolves, it is critical to understand what specific clinical groups are using these resources, how they are doing so, and how those resources can best support health care self-management and disease prevention. This study examined using the internet for health information seeking tasks by individuals with IBD. As expected, frequent internet users were more likely to use the internet for health-related tasks. Our study demonstrates there are a number of factors and complex subgroups that impact the likelihood of individuals with IBD using the internet for information seeking. Future research should further investigate how these factors and groups (eg, women trying to self-regulate care) use the internet for health information and how the use of the internet shapes self-management of their health. Future research should also attempt to identify information design strategies and specific health-related task strategies for this population. In addition, human factors studies should be conducted to identify if and how online resources can support these populations in ways that improve access to information and health outcomes.

## Abbreviations

IBD: inflammatory bowel disease
OR: odds ratio
NHIS: National Health Interview Survey
